# Spontaneous K-Complex Density in Slow-Wave Sleep

**DOI:** 10.1371/journal.pone.0150929

**Published:** 2016-03-10

**Authors:** Md Dilshad Manzar, Mohammad Muntafa Rajput, Wassilatul Zannat, Seithikurippu R. Pandi-Perumal, Ahmed S. BaHammam, M. Ejaz Hussain

**Affiliations:** 1 Centre for Physiotherapy and Rehabilitation Sciences, Jamia Millia Islamia, New Delhi, India; 2 Department of Biomedical Science, College of Health Science, Mizan Tepi University, Mizan Teferi, Ethiopia; 3 Department of Biochemistry, Institute of Home Economics, University of Delhi, New Delhi, India; 4 Somnogen Canada Inc., College Street, Toronto, Canada; 5 The University Sleep Disorders Center, Department of Medicine, College of Medicine, and NSTIP Strategic Technologies Program, College of Medicine, King Saud University, Riyadh, Saudi Arabia; Laboratorio de Neurociencias Moleculares e Integrativas, MEXICO

## Abstract

**Purpose:**

To study spontaneous K-complex (KC) densities during slow-wave sleep. The secondary objective was to estimate intra-non-rapid eye movement (NREM) sleep differences in KC density.

**Materials and Methods:**

It is a retrospective study using EEG data included in polysomnographic records from the archive at the sleep research laboratory of the Centre for Physiotherapy and Rehabilitation Sciences, Jamia Millia Islamia, India. The EEG records of 4459 minutes were used. The study presents a manual identification investigation of KCs in 17 healthy young adult male volunteers (age = 23.82±3.40 years and BMI = 23.42±4.18 kg/m^2^).

**Results:**

N3 had a higher KC density than N2 (Z = -2.485, *p* = 0.013) for all of the probes taken together. Four EEG probes had a higher probe-specific KC density during N3. The inter-probe KC density differed significantly during N2 (*χ*^*2*^ = 67.91, *p* < .001), N3 (*χ*^*2*^ = 70.62, *p* < .001) and NREM (*χ*^*2*^ = 68.50, *p* < .001). The percent distribution of KC decreased uniformly with sleep cycles.

**Conclusion:**

The inter-probe differences during N3 establish the fronto-central dominance of the KC density regardless of sleep stage. This finding supports one local theory of KC generation. The significantly higher KC density during N3 may imply that the neuro-anatomical origin of slow-wave activity and KC is the same. This temporal alignment with slow-wave activity supports the sleep-promoting function of the KC.

## Introduction

The K-complex (KC) was first described almost eight decades ago by a team lead by Loomis [1]. Since then, it has continued to be prominently featured in sleep and neuro-physiological research. Two of the important themes of related research have been KC function and the neuro-anatomy of its generation [2]. KC is a salient waveform of sleep electroencephalography (EEG) and a characteristic feature of the non-rapid eye movement (NREM) sleep stage-2 (N2). KCs appear in both the N2 and N3 stages. KCs are usually not observed in NREM sleep stage-1 (N1) or the rapid eye movement (REM) sleep stages [3]. However, they are visually conspicuous during N2 because of their large amplitudes compared with the relatively low amplitude of the background EEG activity. Their characteristic appearance becomes less obvious in N3 because of the presence of similarly high amplitude delta waves [4, 5]. KCs occur spontaneously during sleep but can also be evoked by sensory stimuli [6]. KC density (KD) represents the number of KCs per min [7–9]. The KC identification and KD documentation have mostly been restricted to N2 [5, 7–11]. There is no documented report of spontaneous KDs in stage N3. However, some studies of the evoked KD have been published [5]. For example, a recent article describes scoring of the KC during stage N3 but does not report the KD or any related measures [12].

There are well-established ethnic differences in sleep and its components. African-Americans have less N3 and higher N2 sleep than Caucasian-Americans [13]. A meta-analysis of fourteen studies found ethnicity-related differences in the proportions of two sleep stages, i.e., N2 and N3. Notably, these sleep stages are also intricately linked to KCs [13]. This may be a logical expression of the ethnic differences in the characteristic EEG features (e.g., KC) of these sleep stages. These differences have potential implications for the definition of normal sleep and its components and associated EEG features in different ethnicities and cultures. Almost all of the documented reports of KD and its topography are focused on American and European populations [5, 10]. Furthermore, no study has assessed spontaneous KD during N3 or KD among Indians.

Previous KD studies have used different numbers of EEG probes [7–9]. Therefore, this study was conducted to estimate the SKD in N3 and to investigate sleep stage differences in SKD among healthy Indian college students using a SKD probe. K-complex epoch density (KCED) (number of epochs with KCs/sleep stage time in min) was used as an index of the temporal spread of KC.

## Materials and Methods

### Study Design and Participants

This is a retrospective study using polysomnographic (PSG) sleep records from the archives at the sleep research laboratory of the Centre for Physiotherapy and Rehabilitation Sciences, Jamia Millia Islamia, India. The PSG sleep recording was performed in compliance with the institutional ethics manual and the Helsinki Declaration. The primary study was approved by the human institutional ethics committee of the Department of Biosciences, Faculty of Natural Sciences, Jamia Millia Islamia (A Central University), New Delhi. The sample was approved for this secondary analysis using electroencephalographic data from the PSG records by the expert committee of the Centre for Physiotherapy and Rehabilitation Sciences, Jamia Millia Islamia. Informed written consent was provided by each volunteer. The PSG records of seventeen young healthy male college students were used. The mean age and BMI were 23.82±3.40 years and 23.42±4.18 kg/m^2^, respectively. The participants had no chronic illnesses. PSG records with very minimal levels of artefacts in the EEG probe (at least during N2 and N3) were selected to investigate KC frequency for the entire sleep duration. KCs were identified manually by an expert polysomnographer according to the KC definition detailed below.

The polysomnographic sleep records had the standard AASM manual-based electrode placements [3]. These records used the standard 10–20 system, three-probe EEG data (F4-M1, C4-M1, and O2-M1). These were further stratified using the post-record data management options of the Quest 32 polysomnograph (RMSIndia, Chandigarh, India) to derive three additional EEG probes (F4-C4, C4-O2, and F4-O2) to study the effect of topographic overlap (covered by EEG probes) on the KD. The EEG electrode impendence values, sampling rate, and bandpass filters were less than 5 kW, 0.3–35 Hz and 256 Hz, respectively. All the epochs of N2 and N3 for each participant were used for KC identification. In total, this study presents KC data for 8918 epochs or 4459 minutes.

### Definition of Measures

KC was defined as an abruptly delineated sharp negative wave immediately followed by a positive component [4, 11] with the following characteristics: (i) a minimal duration of 0.5 sec [3, 9, 11], (ii) an amplitude of at least 75 μv for the negative wave from the EEG-baseline[9, 11], (iii) the presence of a mirror image in the EOG probes [3], (iv) a positive wave amplitude ≥ 50% of the preceding negative wave [4], and (v) the duration of negative wave < the duration of the positive wave [4]. A clearly distinct negative component was essential, but proper care was taken to identify the less-frequent polyphasic waves associated with the onset (or just prior to the onset) of the negative KC sharp wave [4]. Moreover, SKCs were differentiated from delta waves (most notably during N3) using amplitude criteria. The amplitude of KCs is maximized at fronto-central probes [11].

KD was defined as follows: the number of KCs/sleep stage time (min) [7–9]. The K-complex epoch density (KCED) was defined as follows: the number of epochs with KCs/sleep stage time (min). In this study, NREM consists of N2 + N3.

### Statistical Analysis

The Statistical Package for the Social Sciences 16.0 (SPSS Inc., Chicago, IL, USA) was used for data analysis. Descriptive statistics, chi-square, Friedman and Wilcoxon tests were used to determine inter-sleep state and inter-probe differences in KD.

## Results

There were 2311 epochs with KCs in at least one of the six EEG probes during NREM sleep. Overall, 25.92% of the NREM epochs had KCs. Of these, 1289 (55.78%) epochs were of stage N2, and 1022 (44.22%) were of stage N3. The KD expressed as the median (interquartile range) in stages N3 and N2 were 2.02 (3.13) and 1.15 (1.91), respectively. The KCED in NREM, N2 and N3 were 0.37 (0.33), 0.34 (0.41) and 0.50 (0.62), respectively. The correlation between KD and KCED was high in both N2 ((r = 0.985, *p* < .001) and N3 (r = 0.953, *p* < .001) sleep.

## Inter-Sleep Stage Difference

The KD was higher in sleep stage N3 than N2 (*Z* = -2.485, *p* = 0.013) for all the probes taken together. Four of the EEG probes had significantly higher KD in N3 (Tables 1 and 2).

**Table 1:** Inter-sleep stage differences in K-complex density at each EEG probe.

Sleep stage and EEG probes	Z score	*p*
N3/F4/A1-N2/F4/A1	-2.533	= 0.011
N3/C4/A1-N2/C4/A1	-2.343	= 0.019
N3/O2/A1-N2/O2/A1	-0.980	= 0.327
N3/F4/O2-N2/F4/O2	-2.391	= 0.017
N3/F4/C4-N2/F4/C4	-1.189	= 0.234
N3/C4/O2-N2/C4/O2	-2.864	= 0.004

N3 = non-rapid eye movement sleep stage 3, N2 = non-rapid eye movement sleep stage 2.

F4-A1, C4-A1 and O2-A1 are probe placements as per the standard electroencephalographic 10–20 system

**Table 2:** Probe-specific K-complex densities.

EEG probe	N2	N3	NREM
F4/A1	0.35 (0.53)	0.60 (0.94)	1.39 (2.01)
C4/A1	0.11 (0.28)	0.30 (0.31)	0.59 (0.71)
O2/A1	0.00 (0.01)	0.00 (0.01)	0.00 (0.04)
F4/O2	0.35 (0.52)	0.60 (0.88)	1.23 (1.77)
F4/C4	0.20 (0.28)	0.23 (0.60)	0.55 (1.48)
C4/O2	0.16 (0.30)	0.31 (0.30)	0.63 (0.80)

The values are expressed as the median (Inter-quartile range). NREM = N2 + N3, N3 = non-rapid eye movement sleep stage 3, and N2 = non-rapid eye movement sleep stage 2.

F4-A1, C4-A1 and O2-A1 are probe placements as per the standard electroencephalographic 10–20 system.

### Inter-Probe Difference in Each Sleep Stage

We used the Friedman test to assess inter-probe differences in KD during different sleep stages. The analysis showed significant inter-probe differences in KD during N2 (χ2 = 67.91, p < .001), N3 (χ2 = 70.62, p < .001) and NREM (χ2 = 68.50, p < .001). For the post hoc analysis, we used the Wilcoxon test, which showed that all probe pairs except C4/O2-F4/C4 had significant differences in KD during each of the sleep stages studied, i.e., N2, N3, and NREM (Tables 2 and 3).

**Table 3:** Inter-probe differences in K-complex densities during each sleep stage(s).

Probe pair	NREM		N2		N3	
	Z score	*p*	Z score	*p*	Z score	*p*
F4/A1-C4/A1	-3.621	< 0.001	-3.621	< 0.001	-3.621	< 0.001
F4/A1-O2/A1	-3.621	< 0.001	-3.621	< 0.001	-3.621	< 0.001
C4/A1-O2/A1	-3.622	< 0.001	-3.621	< 0.001	-3.622	< 0.001
F4/O2-F4/C4	-3.574	< 0.001	-3.574	< 0.001	-3.575	< 0.001
C4/O2-F4/C4	-0.095	= 0.925	= -0.686	= 0.492	= -0.781	= 0.435
C4/O2-F4/O2	-3.196	= 0.001	= -3.385	= 0.001	= -3.051	= 0.002

N3 = non-rapid eye movement sleep stage 3, N2 = non-rapid eye movement sleep stage 2 and NREM = N2 + N3.

F4-A1, C4-A1 and O2-A1 are probe placements as per the standard electroencephalographic 10–20 system.

### Distribution Across Sleep Cycles

**Fig 1:**
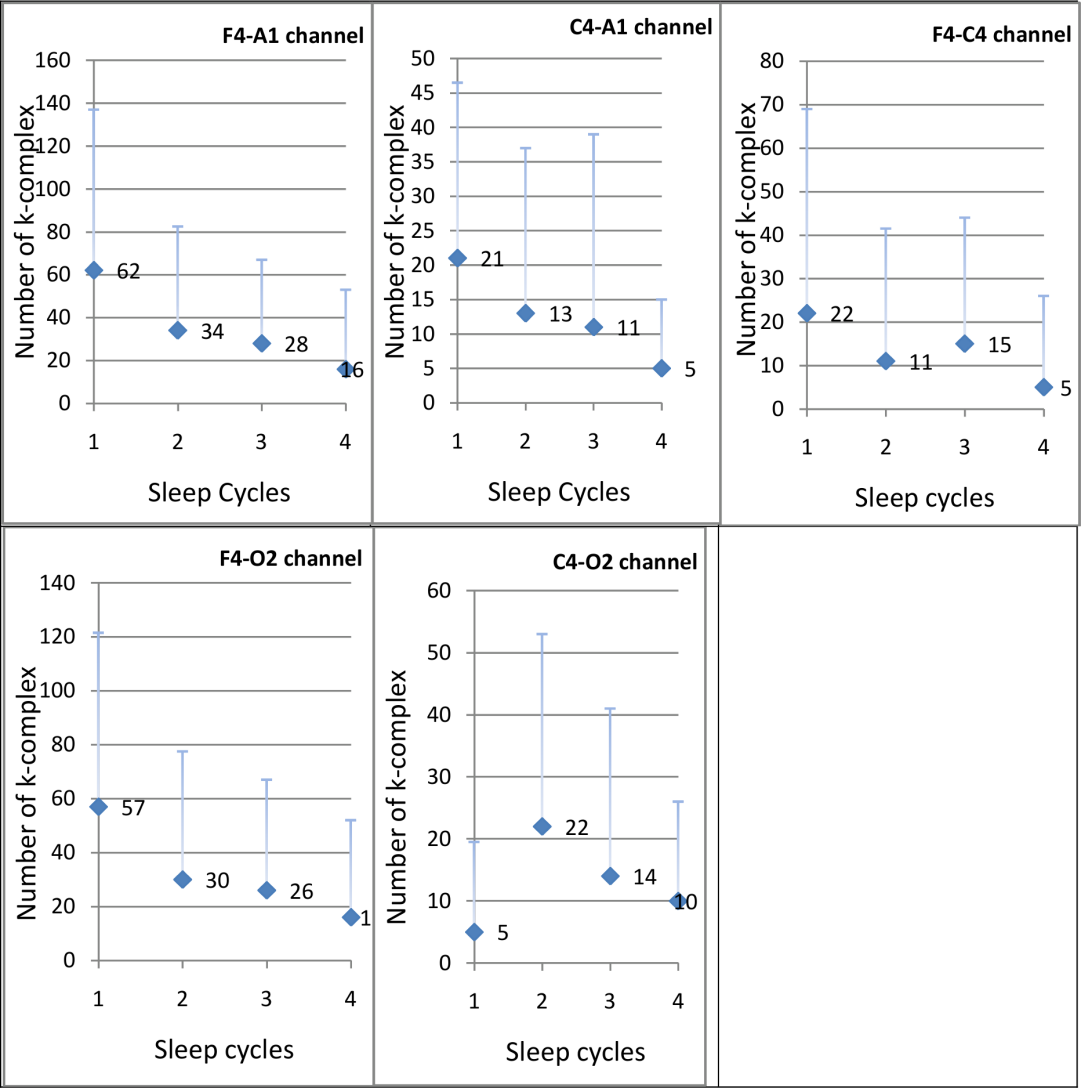
Trend of probe-specific K-complex numbers across sleep cycles. Each panel represents the probe-specific K-complexes across different cycles. The Y-axis shows the means and standard deviations of absolute numbers of K-complexes. The X-axis shows the first four cycles of the sleep stages.

The percent distribution of KCs decreased uniformly as the sleep cycle progressed: 37.34%, 28.37%, 20.61%, and 13.68% in sleep cycles 1–4, respectively. The fifth cycle had very few data and was, thus, not included in the analysis. This decreasing trend was also observed in the number of KCs across all EEG probes individually (Fig 1). Probe O2-A1 was not included in Fig 1 because it recorded very few KCs.

## Discussion

This study provides baseline data for spontaneous KD in N3, including probe-specific KD and KCED for N2 and N3. Moreover, it is the first study to document the distribution of KD and KCED in Asian Indians. This is important given that sleep stages have been shown to depend on ethnicity. The component waves of the auditory-evoked KCs, e.g., N550 and P900, have differential scalp distributions. The polarity of N550 is inverted at the mastoid and inferior parietal regions. The decline pattern of the N550 is sleep stage dependent over the parietal and posterior–inferior areas [8]. Therefore, future studies with a more elaborate and comprehensive number of EEG channels should facilitate further exploring and validating the findings of this study. More than a quarter of NREM epochs had KCs. N2 had a greater absolute number and percentage of epochs with KC. However, N3 had significantly higher KCED than N2 (Z = -2.722, *p =* .006). This is understandable given that the amount of N2 is usually higher (most often nearly twofold higher) than N3 in healthy young adults. Similar to earlier reports, the percent distribution of KCs decreased along the sleep cycles [14]. This decreasing trend was also observed in the KCs (absolute number) across individual EEG probes along the sleep cycles (Fig 1).

KCED was introduced to increase vibrancy in the reporting of KD. It has a theoretical range of values between 0–2. The availability of KCED and KD together provide a better overview of the temporal alignment and distribution of KC along the entire sleep/sleep stage span.

In our data, both KCED and KD are higher for N3, which means that the frequency of KCs is higher in N3. Additionally, they are also relatively widespread. There are some practical issues with how KD is used in the literature. Counting KCs requires pooling of all KCs observed via all probes during the entire duration of the stage/time investigated. However, the absolute number of KCs depends on the number and topography of the EEG probes used [7, 11, 15]. No standard practice regarding the number and topography of the probes used has been established, and as a result, the standard 10–20 EEG montage was employed [7–9]. In such a situation, comparison between KDs from different studies is meaningless. To overcome this problem, we introduced the use of probe-specific KDs in addition to the sleep stage-specific KDs in this study.

The finding of inter-probe differences in KD during a particular sleep stage (Tables 2 and 3) is similar to earlier reports [7, 15]. However, the documentation of the statistical significance for these differences may represent an important finding. Similar to previous reports, the KD had fronto-central topographic dominance and exhibited the following pattern of significant differences: frontal>central>occipital [7, 10, 15–17]. The topographical evidence from the intracranial distribution of the human KC in refractory epilepsy patients is strikingly similar [17]. A recent work on evoked (acoustic, tactile and visual) KCs reported a maximal detection rate in the fronto-central areas of the N550 and P900 components of the KC [16]. Our findings provide additional evidence that fronto-central dominance is also observed in spontaneous KDs during N3 sleep (Tables 2 and 3). This is an important observation for the nearly eight-decade-long debate regarding the neuro-anatomical origin of the KCs [1, 16]. Although the present data cannot provide direct evidence of which brain regions are involved in KC generation, the consistency of the topographical trend of KD irrespective of sleep stage supports the neuro-anatomical locale theory of KC generation. N3 had significantly higher KD at four of the six EEG probes (Tables 1 and 2). This finding could be interpreted as follows: KCs start appearing prior to SWS but subsequently achieve maximal KD as SWS increases. This behavior lends support to the research suggesting the same neuro-anatomical origin for both KC and slow-wave activity [10, 18]. Furthermore, as suggested earlier, this concurrence of KC and SWS may indicate that KCs exert a sleep-protecting/promoting effect [8, 10, 11, 18].

The EEG amplitudes at each probe are measured in reference to the electrical activity of the mastoid on the opposite side of the head, i.e., F4 = F4-M1. Thus, the amplitude of the electrical activity at M1 is subtracted from those at F4. The electrical activity at M1 is the electrical activity at M1 minus the electrical activity at two reference points located at the nasion. F4/C4 represents the electrical activity of C4 subtracted from F4. In the same manner, C4/O2 and F4/O2 were also used to investigate topographic overlap. The post-recording-derived EEG probes F4/C4, C4/O2 and F4/O2 were used to understand the effect of spatial overlap on the KD. The KD in the F4/O2 probe was nearly the sum of the KDs of F4/C4 and C4/O2 (Table 2). The area of the brain region represented by F4/O2 is the sum of the region covered by F4/C4 and C4/O2. This spatial summation reflecting the summation of the KDs is an interesting finding. The minute discrepancy can be accounted for by the fact that using EEG leads as a reference instead of M1 would have reduced the amplitude of the waves. More specifically, in the context of KC detection, probe F4/C4 would have been affected more severely than F4/O2 and C4/O2 because of the fronto-central dominance of the K-complex amplitude [11]. This effect would have artificially reduced the KDs in F4/C4 and may explain the non-significant difference in the KDs between F4/C4 and C4/O2 despite the fronto-central dominance of the KDs.

KCs are routinely induced in research using sensory stimuli, such as acoustic, tactile and visual stimuli [9, 15, 16, 18]. The sleep-promoting effect of KC [8, 11, 18] makes this a prospective route for physical agent-based sleep management. The probe-specific KD will facilitate precisely estimating the treatment effect. The results of this study also provide objective indices (KD and KCED) to standardize dose responses in evoked KC experiments.

Previous reports have typically used selected segments of the N2 stage record for KD estimation [7]. This approach may result in over/under-reporting because KD continuously decreases across sleep cycles. In the case of biased selection of segments from initial sleep cycles, KD may be over-reported, while biased selection of segments from late cycles may lead to under-reporting KD [14]. In the present study, the entire NREM (N2+N3) EEG record was analyzed for KD estimation. Therefore, the results are more likely to be free from the bias of under/over-reporting.

This study has several limitations. For example, relatively few EEG probes were used because the records were not traditional EEG records but were, instead, PSG records. Hence, future studies employing higher numbers of EEG probes should be used to investigate spontaneous KD and KCED during N3. The issues of gender exclusivity and the narrow age range of the participants should also be addressed in future studies.

This probe-specific density/frequency may be used to report other characteristic EEG features, e.g., sleep spindles. Future studies should investigate and standardize the normative and clinical values of the probe-specific KD and KCED. The inter/intra-scorer reliability of probe-specific KD and KCED should also be examined.

## Conclusions

The N3 stage has higher KD and KCED than the N2 stage. The topography of the fronto-central dominance of spontaneous KD is independent of sleep stage, i.e., N2 and N3. The use of probe-specific KD and KCED is recommended for reporting the frequency/density of the K-complex. Future research should specifically investigate the ethnic dependence of KD and KCED.

## Supporting Information

S1 Fig Certificate of professional English language editing. (PDF)

S1 Table Complete data file in excel format. (XLSX)
