# Supplementary material for: Spontaneous K-Complex Density in Slow-Wave Sleep
Source: PLoS One. 2016 Mar 10;11(3):e0150929. doi: 10.1371/journal.pone.0150929 (PMC4786157; doi:10.1371/journal.pone.0150929)
Supplement: S1 Fig — (PDF) [file pone.0150929.s001.pdf]

nature publishing group language editing
Premium science and medical editing

NPG Language Editing Editorial Certification

This is to certify that the manuscript titled Spontaneous K-complex density in Slow wave sleep was edited for English language usage, grammar, spelling and punctuation by one or more native English-speaking editors at NPG Language Editing. The editors focused on correcting improper language and rephrasing awkward sentences, using their scientific training to point out passages that were confusing or vague. Every effort has been made to ensure that neither the research content nor the authors' intentions were altered in any way during the editing process.

Documents receiving this certification should be English-ready for publication; however, please note that the author has the ability to accept or reject our suggestions and changes. To verify the final edited version, please visit our verification page. If you have any questions or concerns over this edited document, please contact NPG Language Editing at support@languageediting.nature.com.

Manuscript title: Spontaneous K-complex density in Slow wave sleep

Authors: Md Dilshad Manzar1¶, Rajput Mohammad Muntafa2¶, Wassilatul Zannat1, Seithikurippu R. Pandi-Perumal3, Ahmed S. BaHammam4, M Ejaz Hussain1, *

Key: F9AB-1614-9CD0-B7EA-DC2F

This certificate may be verified at **languageediting.nature.com/certificate**.

NPG Language Editing is a service from Nature Publishing Group, the publishers of Nature and purveyors of scientific and medical excellence in print and online.

NPG Language Editing comprises a network of more than 900 editors with a range of scientific, technical and medical backgrounds. All our editors must meet strict selection criteria. We require that each editor has completed or is attending a graduate program at one of the top universities in the USA. He or she must have proven written communication skills and undergo a comprehensive training period under the instruction of a managing editor. All editors are native English speakers.

Uploaded articles are reviewed by an editor with relevant scientific background. To ensure we can meet the needs of researchers in all scientific fields, we continually recruit editors to represent growing and new disciplines. Our senior editors quality-assess each edited manuscript before it is returned to the author. They ensure that the high standards to which NPG Language Editing aspires are maintained.
